# Mechanical Response of Carbon Composite Octet Truss Structures Produced via Axial Lattice Extrusion

**DOI:** 10.3390/polym14173553

**Published:** 2022-08-29

**Authors:** Pritam Poddar, Mark Olles, Denis Cormier

**Affiliations:** Industrial and Systems Engineering, Rochester Institute of Technology, Rochester, NY 14623, USA

**Keywords:** octet truss, lattice structures, fused filament fabrication, engineered cellular materials

## Abstract

Engineered lattice structures fabricated via additive manufacturing (AM) technologies are of great interest for many applications that require high strength and/or stiffness with minimum mass. This paper studies a novel axial lattice extrusion (ALE) AM technique that greatly enhances mechanical properties of polymeric lattice structures. When the novel ALE process was used to produce 84 mm × 84 mm × 84 mm octet truss lattice samples using fiber reinforced ABS, a total of 219,520 polymer interfaces in the lattice beams were eliminated relative to the conventional 3D printing alternative. Microscopic examination revealed near perfect alignment of the chopped carbon fibers with axes of the cylindrical beams that make up the lattice structure. The greatly enhanced beam quality with fiber reinforcement resulted in excellent mechanical properties. Compression testing yielded an average relative compressive strength of 17.4 MPa and an average modulus of 162.8 MPa. These properties rate very strongly relative to other published work, and indicate that the ALE process shows great potential for fabrication of high-strength, lightweight, large-scale, carbon-fiber composite components. The paper also contributes a modeling approach to finite element analysis (FEA) that captures the highly orthotropic properties of carbon fiber lattice beams. The diagonal shear failure mode predicted via the FEA model was in good agreement with experimentally observed results.

## 1. Introduction

**Figure 1:**
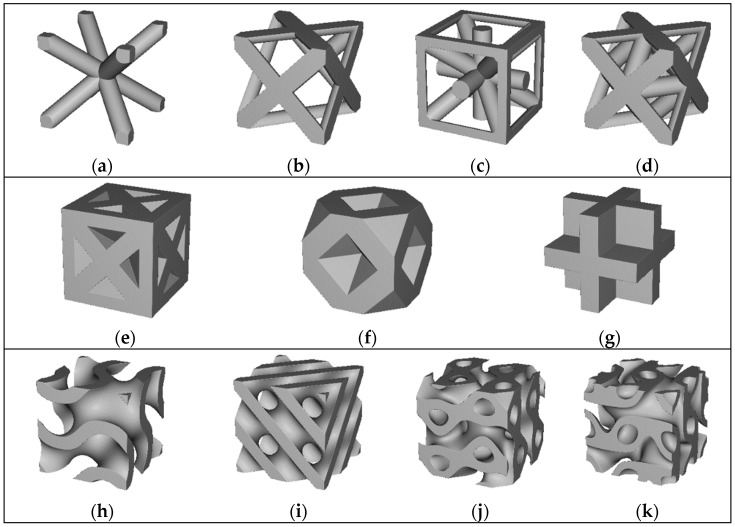
Open cell beam-based lattice structures (**a**–**d**), closed-cell foam based structures (**e**–**g**), and triply periodic minimal surface (TPMS) structures (**h**–**k**); (a) body centered cubic unit cell; (**b**) face centered cubic unit cell; (**c**) isotruss unit cell; (**d**) octet truss unit cell; (**e**) body centered cubic foam; (**f**) face centered cubic foam; (**g**) simple cubic foam; (**h**) gyroid TPMS unit cell; (**i**) diamond TPMS unit cell; (**j**) SplitP TPMS unit cell; (**k**) Lidinoid TPMS unit cell.

Engineered lattice structures have been of keen interest to the metal additive manufacturing (AM) research community for many years. They have been used to reduce weight in aerospace applications [1], provide porous in-growth surfaces in bone implants [2], and reduce the amount of material and time needed to fabricate parts [3]. Lattice structures used in these applications are typically constructed as three-dimensional (3D) arrays of a repeating unit cell geometry, or “building block”, such as those seen in Figure 1. Figure 1a–d show four unit cells that form open-cell lattices when patterned into 3D arrays. Figure 1e–g show three unit cells that form closed-cell lattices when patterned into 3D arrays. Lastly, Figure 1h–k show four types of triply periodic minimal surface (TMPS) unit cells that have unique properties in which the mean value of surface curvature is zero at all points.

From visual examination of the unit cells in Figure 1, it is readily apparent that large arrays of repeating lattice unit cells cannot easily be fabricated via traditional manufacturing methods such as molding, casting, machining, or forming. The overwhelming majority of lattice structures are fabricated via additive manufacturing (AM) methods. Despite the growing use of metallic engineered lattice structures, industrial adoption of polymer lattice structures has been limited. This can be largely attributed to relatively poor mechanical properties of polymeric lattice structures compared with their metallic counterparts. The primary motivation for this research, therefore, is to improve mechanical performance of polymer engineered lattice structures using a novel AM technique with carbon fiber composite feedstock material.

One potential way to enhance mechanical properties of polymeric lattice structures is to consider use of carbon fiber reinforced polymer matrix (CFPM) composite materials. CFPM’s are increasingly being specified in the transportation industry as lighter weight replacements for some metallic materials. Weight reduction in transportation applications improves energy efficiency, reduces fuel consumption, and reduces CO_2_ emissions.

**Figure 2:**
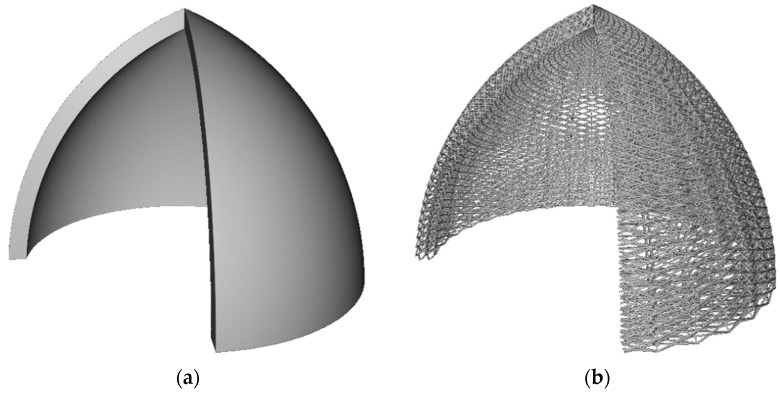
(**a**) Section view of solid dome shape; (**b**) Section view of conformal lattice dome shape.

Although most research involving engineered lattices use AM to fabricate the structures, one of the simplest ways to construct engineered lattices is to cut 2D pieces from sheet material, and to then assemble those pieces into 3D structures. This is analogous to how 3D puzzles are constructed. For example, Jenett et al. [4], Xu et al. [5], and Dong and Wadley [6] each describe manual assembly approaches to fabricating cellular structures. These research groups use low cost methods such as waterjet machining, laser cutting, or CNC machining, to cut 2D patterns from sheet-based material. The 2D shapes are manually assembled with interlocking joints, and then bonded using adhesives, brazing, sintering, or similar methods. The significant advantages of this approach are its simplicity and relatively low cost. Dong and Wadley’s work is also noteworthy in that it demonstrated the ability to cut carbon fiber composite beams from sheet material, thus providing excellent strength and stiffness in the assembled lattice structures. These methods show excellent potential for use in low volume production. However, drawbacks include high labor content as well as potentially high material wastage (scrap) when cutting 2D components out of expensive carbon fiber composite sheets/plates. Furthermore, they are not well suited for producing lattice structures out of non-cubic shapes, such as the dome structure shown in Figure 2a. Figure 2b shows a conformal lattice structure in which the lattice unit cell bounding boxes are not cubic, as shown in Figure 1, but rather they are morphed to conform (flow) with the shape of the non-planar surfaces they are bound by.

As previously mentioned, the most widely used polymer AM processes to fabricate lattice structures fall into four categories. Vat polymerization methods, such as stereolithography (SLA), uses a scanning laser or projected image to photopolymerize selected regions of a layer of a (typically) acrylate or urethane photopolymer resin. Then, a new layer of resin is spread onto the first layer, and a second layer pattern is cured. The process repeats itself one layer on top of another until the part is complete. Dar et al. [7] used the projection SLA method to fabricate BCC lattice structures, and Ge et al. [8] present a review of research using a micro-projection SLA with resolution capabilities down to 2 μm/pixel. Vat polymerization can be used to fabricate micro and even nano-scale lattice structures with incredible feature resolutions [9]. Li and Shen [10] present a very interesting approach to designing 3D printed lattice structures in which the beam thickness in face-centered cubic (FCC) unit cells was locally increased or decreased based on FEA simulations. In areas with high stress, the beam thicknesses were increased to strengthen those regions. In low stress areas, the beam thickness were decreased in order to reduce mass. This resulted in heterogeneous lattice structures. The design method was validated using the stereolithography 3D printing process and 3-point bend tests. Although this research study did not use composite polymer matrix composite material, the design method would be amenable to use with the Axial Lattice Extrusion 3D printing technique described in this paper. The potential combinations are described in the Future Research section of the paper.

Although vat polymerization is extremely well suited for nano/micro-scale lattice structures, they are not as well suited for producing physically large lattice structures needed for transportation applications. Large vats of photopolymers would be extremely costly, and would be difficult to shield from oxidation and unwanted UV curing from stray light each time the machine is opened. Postprocessing of these parts also requires submersion and rinsing in solvent baths to remove uncured resin from the surfaces of the parts. That would be a challenge for extremely large parts. Lastly, the need to remove uncured resin from the beams of lattice structures would be problematic for parts whose surfaces are made of solid skins that encapsulate the internal lattice structure (i.e., removal of trapped uncured resin would be a challenge).

The second mainstream polymer AM process that may be used to fabricate lattice structures is material jetting. Polymer material jetting is typically done via layerwise inkjet printing and UV curing of acrylate or epoxy photopolymers. AM via inkjet material deposition is intriguing in the sense that (1) hundreds of parallel nozzles jetting material at typically 2 kHz or greater frequencies allows parts to be built at higher speeds than most other polymer AM processes, and (2) the use of multiple inkjet heads allows multiple materials with different properties to be deposited. In the same way that cyan, yellow, and magenta inks can be combined in different proportions to produce desired pixel colors in document printing, precise control over Shore hardness within a 3D printed part can be achieved by blending rigid and soft photopolymers in the desired proportion at each voxel (3D pixel) location via inkjet printing. For example, Kozior and Kundera [11] used a Stratasys Eden material jetting machine to print 13 mm × 13 mm × 6.3 mm and 26 mm × 26 mm × 12.6 mm cellular structures using blends of rigid and soft materials. The samples were compressed in order to assess stress relaxation of the blended material structures as a function of time.

As is the case with vat polymerization, using material jetting methods to produce large scale high strength lattice structures is a challenge. In order for 3D inkjet printing to work with lattice structures, the void space within the lattice structure must be completely filled with a sacrificial support material that is removed after printing is complete. This support structure must be removed via post-processing, and that is exceedingly difficult if the parts have solid skins (surfaces) on them. Furthermore, the photopolymers must have very low viscosity (typically <10 cP) and precisely controlled surface tension (typically ~30 dynes/cm) to be compatible with inkjet printing. These photopolymers are very costly on a volumetric basis, which makes fabrication of large parts impractical. For these reasons, photopolymer material jetting has not been widely studied for production of polymer lattice structures.

Polymer powder bed fusion (PPBF) is the third mainstream AM process to be considered for making polymer lattice structures. With PPBF, a layer of thermoplastic powder is spread out, and radiant heating is used to preheat the powder bed to prevent thermal distortion during processing. A laser is then selectively scanned over regions of the powder bed to be melted. A fresh layer of powder is spread on top of the first layer, and the process is repeated layer-by-layer until the part is complete. The process is largely limited to for with semi-crystalline thermoplastics due to the need for powder scanned by the laser to rapidly melt, flow out and merge with previously densified material, and then rapidly solidify. The melt viscosities of amorphous thermoplastics are generally too high to flow out and properly densify for use with SLS [12]. Polyamide (PA12) is by far the most commonly used thermoplastic with polymer PBF.

Cobian et al. [13] printed small 30 mm × 15 mm × 15 mm “octet like” PA-12 lattice cubes with PPBF. Tension, compression and nanoindentation testing was performed on the samples. The authors concluded that the PPBF lattice structures were highly anisotropic, and that load direction was an important consideration when designing structures. Jin et al. [14] propose a non-uniform lattice design approach in which the diameters of individual struts can be thickened where needed to better withstand expected mechanical loading using a topology optimization approach. The proposed method was demonstrated using PA-12 with a PPBF machine. Neither of these papers used fiber reinforced feedstock material, and both used relatively small sample sizes.

Despite the advantages of PPBF, commercially available machines are generally limited to modest build volumes on the order of 700 mm × 350 mm × 350 mm. This limits their utility for producing large-scale lattice structures. One reason for this is the need to preheat the powder bed to reduce/prevent thermal distortion of the part. Unused powder from each print job goes through heating and cooling cycles that significantly degrade the powder’s material properties. Unused powder can be blended with virgin powder, however, repeated thermal cycles will adversely affect mechanical properties. It is noted that metallic PBF processes (e.g., laser or electron beam PBF) have been the dominant method used to produce metallic lattice structures to date [15,16,17,18]. Aluminum and titanium alloys most typically used to produce these lattices have densities of ~2.7 and 4.2 g/cm^3^, respectively. Likewise, the cost of their powders are in the $100/kg and $275/kg range, respectively. By way of comparison, carbon fiber composite feedstock materials of interest in this study have densities of ~1.2 g/cm^3^ and prices of ~50/kg (exact values depend on the specific feedstock of course). Give the 2X–4X higher density and 2X–5X higher prices of metal powders compared with carbon fiber polymer matrix composite feedstocks, the aim of this paper is to explore lower cost and lighter weight polymer composite lattice fabrication. Metal PBF of lattice structures is therefore not covered any further here.

By far the largest category of mainstream polymer AM processes is fused filament fabrication (FFF). With this process, thermoplastic filament is extruded through a nozzle onto a moving substrate to lay down material in a desired geometric pattern. The process is repeated layer-by-layer until the 3D part is completed. Unlike most other polymer AM processes, FFF is very well suited for the fabrication of large parts, such as those of interest in this work. For example, Post et al. [19] used FFF to fabricate a full-scale boat hull that was more than 10 m long. Naboni et al. [20] used a similar process to fabricate a pavilion structure covering an area of 36 m^2^. Another advantage of FFF is that low cost commodity thermoplastic materials used in conventional plastic injection molding can be used. These include thermoplastic materials reinforced with chopped carbon fibers to enhance mechanical properties.

Despite the advantages of FFF, the use of FFF to produce lattice structures for practical applications has been severely limited. This can be largely attributed to the relatively poor interlayer bonding associated with FFF. Unlike metal AM processes in which interlayer metallic bonding is generally quite good, interlayer bonding in FFF processes is known to be poor [21]. This poor bonding can be explained using reptation theory which is often used to characterize bond strength between polymer interfaces [22]. This theory holds that the bond strength at an interface (i.e., interfaces between printed layers in an FFF part) is heavily influenced by (1) the temperature of the polymer(s) when the interface is created, (2) the time that the polymers are maintained above the glass transition temperature, and (3) the amount of pressure applied at the interface during processing. This unfortunately results in conflicting objectives for FFF when large scale parts are to be produced. Unlike injection molding, thermoplastic material extruded from a nozzle is not constrained within a cavity. It is therefore difficult to apply high pressure during FFF to improve interlayer bonding. That leaves temperature and time as the primary controllable factors. If one chooses to 3D print at very low speeds and elevated temperature in order to produce high strength bonds between printed layers, the time needed to produce physically large parts becomes unacceptably long. If one increases print speed to reduce build times, then very poor interlayer bond strengths are to be expected.

**Figure 3:**
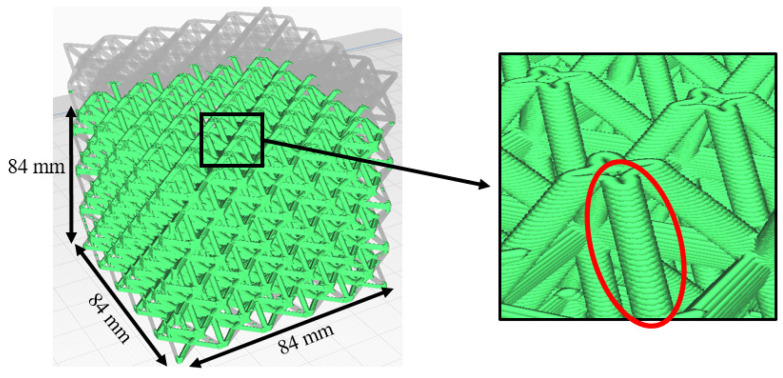
3D lattice structure (left), and close-up view showing how individual cylindrical beams are produced as the result of a lamination of dozens of layers with small cross-sectional areas.

These challenges are exacerbated when using FFF to print lattice structures. Figure 3 illustrates a 3D lattice structure that is comprised of an array of vertical, horizontal, and diagonal cylindrical beams. When this structure is sliced into planar layers of material that get printed via conventional FFF, one can clearly see in the zoomed-in inset figure that each beam is produced as the result of dozens of vertically stacked small areas of extruded thermoplastic material. In the specific example shown in Figure 3, the diagonal beams have a vertical height of 6 mm, and each printed layer has a thickness of 0.15 mm. Each beam therefore has 6 mm ÷ 0.15 mm = 40 polymer interfaces between printed layers. The structure as a whole in Figure 3 has 5488 beams. Multiplying the number of beams by 40 polymer interfaces per beam, one arrives at 219,520 polymer interfaces in a small lattice cube that measures 84 mm × 84 mm × 84 mm. When this structure undergoes mechanical loading, there are literally hundreds of thousands of relatively weak polymer layer interfaces where cylindrical beams can fail. For parts at the scale of automotive, rail, marine, or airplane size scales, the number of polymer interfaces would be in the millions.

Although lattice structures printed via FFF have inherent difficulties stemming from hundreds of thousands or more weak interlayer bonds, there is considerable literature devoted to FFF lattice printing with different materials, geometries, and design methodologies. Abusabir et al. [23] studied quasi-static and visco-elastic responses of simple cubic lattice structures 3D printed using polylactic acid (PLA) and acrylonitrile butadiene styrene (ABS) polymers. The authors concluded that plate lattice geometries were ideal for applications requiring high stiffness and creep resistance, whereas a shell-based cubic lattice structure was preferred for energy damping performance when quasi-static loading conditions are expected. All samples printed in the study used the traditional layerwise FFF printing process, and PLA and ABS polymers without fiber reinforcement were used.

Basurto-Vazquez et al. [24] studied the energy absorbing performance of 3D printed polyethylene terephthalate glycol (PET-G) 2D cellular honeycomb structures fabricated using different orientations and infill densities. Compression testing revealed that 3D printing using 100% infill with an upright honeycomb orientation produced the best energy absorbing performance. All samples printed in this study used the traditional layerwise FFF printing process, and the PET-G material did not include fiber reinforcement.

Whereas the majority of engineered lattice publications deal with open-cell beam structures, Lin et al. [25] studied wall-based lattice structures known as triply periodic minimal surfaces (TMPS). In recognition of the fact that numerical finite element analysis of large lattice structures can be computationally expensive, this study compared numerical FEA simulation results for a 3-point bend test with results predicted by purely analytical methods. The results differed by approximately 21%, which suggests that analytical methods may be suitable for initial design feasibility studies, but that it may not be accurate enough to guide final detailed design decisions.

None of the above mentioned FFF lattice papers made use of fiber reinforced composite feedstock material in an attempt to strength the structure. However, Cuan-Urquizo et al. [26] examined the effect of 2D infill pattern geometry and infill density on bending stiffnesss using a wood PLA composite material. Specifically, 125 mm long × 25 mm wide × 6 mm thick three point bend test coupons were fabricated using hexagonal and star 2D infill patterns were printed with 20%, 30%, 40%, and 50% infill densities. The study concluded that the hexagonal infill pattern resulted in higher stiffness values with low infill percentages, whereas the star infill pattern resulted in higher stiffness values with higher infill percentages. Although this study used a composite material to produce FFF infills, the use of beam-based lattices was not considered.

Della Ripa et al. [27] studied mechanical performance of several different lattice unit cell geometries that were printed using the traditional layerwise FFF process. This study used carbon fiber reinforced nylon as the feedstock material. The octet truss lattice geometry was found to have superior specific energy absorption (SEA) properties under quasi-static conditions. This paper also presented a simplified FEA simulation model that used 1D elements to quickly approximate behavior of the beams that make up the lattice structure. This publication used 3 × 3 × 3 unit cell arrays. It is common practice in the lattice design literature to use 5 or more unit cell replicates in each direction order to obtain reasonably accurate mechanical properties that are not skewed by behavior of unit cells at the outside (edge) faces of the cube [28]. The paper does not describe the failure modes during testing in terms of where and how the structures failed.

Boursier Niutta et al. [29] extended the Dell Ripa et al. study by focusing exclusively on the octet truss lattice structure. Small octet truss unit cell arrays having 2 × 2 × 2, 3 × 3 × 3, and 4 × 4 × 4 replicates were printed using the conventional FFF process. Specific energy absorption values for a range of unit cell sizes and beam diameters were studied. The study also presented a computationally efficient finite element simulation approach with satisfactory agreement between simulated and experimental results. This study used traditional layerwise FFF, and the number of unit cell replication along the X, Y, and Z axes (2, 3, or 4) would ideally be higher to avoid the previously mentioned edge effects.

One aspect off all of the recently published FFF lattice printing work reviewed above is that they all use traditional layerwise FFF that produces large numbers of weak interlayer bonds within each strut as illustrated in Figure 3. Figure 3 illustrates the conventional layer-by-layer FFF process by which a lattice structure would normally be printed. If it were possible to instead print each cylindrical beam as one smooth continuous extrusion, then all of the interlayer bonds within each beam would be eliminated. At the same time, it is well known that the mechanical properties of carbon fiber composites are strongly dependent upon the fiber orientation [30]. For engineered lattice structures, the maximum bending stiffness and strength in tension would occur if the fibers could be aligned with the longitudinal axis of each cylindrical strut. It is well known that chopped fibers become strongly aligned as they exit the extrusion nozzle in the FFF process [31,32,33]. Thus if the cylindrical beams were extruded in continuous move from the base of the strut to the tip of the strut, and if the extruded material contained chopped reinforcing fibers, then one would expect those fibers to be aligned with the axis of the strut. The remainder of this section explores these concepts that form the crux of this paper.

**Figure 4:**
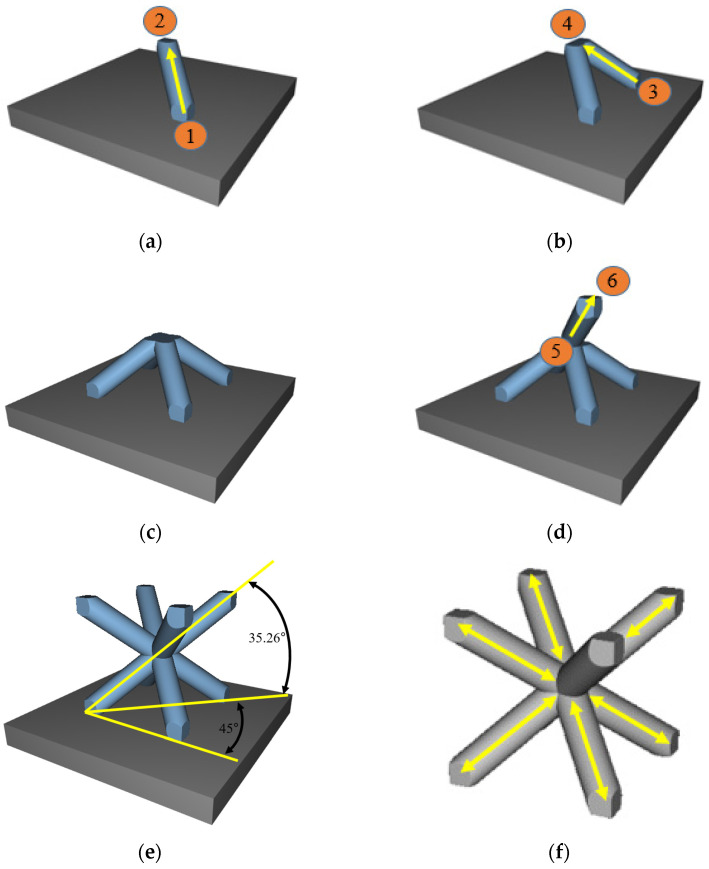
(**a**) Nozzle start and end positions for printing the 1st beam of a BCC unit cell via ALE; (**b**) start and end positions for printing the 2nd beam; (**c**) completed beams for the bottom half of the unit cell; (**d**) start and end nozzle positions for printing the 1st beam on the top half of a unit cell; (**e**) completed unit cell that includes beam orientation angles; (**f**) lattice unit cell illustrating expected orientation of carbon fibers with the longitudinal axis of each cylindrical beam in the BCC unit cell.

This paper seeks to explore the potential of FFF to produce high strength polymer composite lattice structures. The objective is to produce lattice beams that are smooth and continuous, and in which the fibers within each beam align with the longitudinal axis. In order to do this, the traditional layer-by-layer FFF fabrication approach is abandoned in favor of an Axial Lattice Extrusion (ALE) approach [34]. With ALE, each vertical or diagonal strut within a lattice structure is produced in one single continuous upwards extrusion motion rather than being printed as a lamination of many small area patches. The process is illustrated in Figure 4. First, the FFF extrusion nozzle starts at the base of the first beam (Point 1) and continuously extrudes in a diagonal upward motion to Point 2 (Figure 4a) to extrude the first beam in one single movement. Material extrusion from the nozzle is stopped, and the extrusion head moves to the base of the second beam (Point 3 in Figure 4b). Material is then extruded while the nozzle moves from Point 3 to Point 4 (Figure 4b). Figure 4c shows the 3rd and 4th struts on the bottom half of a simple unit cell after printing. To print the first strut in the top half of this unit cell, the extrusion head starts at Point 5 and extrudes continuously to Point 6 (Figure 4d). The remaining three beams in the upper half of this unit cell are produced in similar fashion to produce a complete unit cell as seen in Figure 4e. Finally, Figure 4f shows the expected fiber alignment with each cylindrical beam. The orientation angles of the beams in Figure 4e are provided as a convenience for the reader. For any size cube with a side length of *L*, the angle (α) of any beam that connects opposing corners of the cube is:

(1) $$\alpha =\tan^{-1}\left(\frac{L}{\sqrt{2L^{2}}}\right)=35.26{}^{\circ}$$

In order to prevent the extruded material from sagging during the unsupported upwards motion, a gentle stream of air or nitrogen gas is blown onto the extruded beams to rapidly cool and solidify the extruded material. While Figure 4 illustrates the ALE method for printing one unit cell, the process is readily repeated as many times as needed to print 3D repeating arrays of unit cells. The continuous extrusion movement that starts at the base of each beam and ends at the upper tip of each beam is ideal in the sense that (1) it produces a smooth beam that completely eliminates all weak interlayer bonds within the beam, and (2) the chopped fibers will theoretically align with the axis of the strut during extrusion, thus providing considerable tensile strength and bending stiffness.

**Figure 5:**
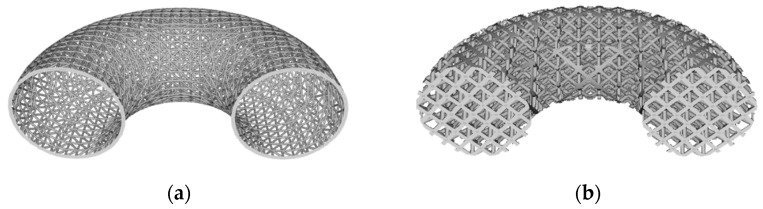
(**a**) Surface lattice, and (**b**) volume lattice.

Variations on the ALE deposition strategy have been explored by a very small number of researchers. Most published ALE related research has focused exclusively on printing just the exterior wireframe surface of an object via ALE [35,36]. Figure 5a shows a surface lattice where the surface of a 3D volumetric shape is covered with a lattice structure, but the inside is hollow. 3D printed surface lattices are motivated by the desire to quickly prototyping the shape of an object, however, they are not generally intended for structural applications. Of greater interest for this work is use of FFF to produce polymeric engineered truss lattice structures that occupy the full 3D volume of a part. A volumetric lattice is shown in Figure 5b.

Among the published work, Liu et al. [37] is one of the few to specifically describe a volumetric ALE deposition strategy using carbon fiber reinforced thermoplastic feedstock material. In this work, sandwich core panels were made using FFF to print one, two, and three layer pyramidal lattice structures that were then epoxied to the face sheets. PLA reinforced with continuous carbon fiber was the feedstock material. Quasi-static compression tests were performed using BCC lattice structures to assess performance as a function of length of the extruded beams. The maximum achieved compressive strength modulus values were 1.24 MPa and 27.70 MPa, respectively. The fact that this study used continuous carbon fiber reinforcement is quite noteworthy, although the researchers reported some complications with lattice printing.

Among the polymer AM processes that have been used to fabricate lattice structures, FFF stands out as the process that is best suited to produce large scale lattices needed for transportation applications that will most benefit from the high strength and stiffness to weight ratios. However, improvements are needed in the traditional layerwise FFF process to eliminate hundreds of thousands to millions of relatively weak polymer interfaces. The remainder of this paper is dedicated to a feasibility study on use of the ALE approach to produce octet truss lattice structures such that (1) the polymer interfaces within the beams are eliminated, and (2) fiber reinforcements are aligned with the axis of the beams. Finite element modeling that captures the highly orthotropic nature of the fiber reinforced lattice structures is of value for engineering design and analysis purposes, hence a modeling approach and experimental validation are also presented. Experimental testing is used to assess performance of the preliminary ALE fiber reinforced lattices and to guide recommendations for future research.

## 2. Materials and Methods

Engineered lattice structures produced using an ALE deposition strategy with carbon fiber reinforced feedstock material will have highly orthotropic material properties. This is the result of a high degree of fiber alignment with the axis of each strut. Numerical simulation to analyze and predict mechanical performance of orthotropic truss lattices is not trivial due to the numerous different beam orientations and node bonds. Section 2.1 describes the setup of a numerical simulation model that captures the orthotropic nature of the carbon reinforced struts in the octet truss lattice structure. Section 2.2 then describes the fabrication of samples used to experimentally validate the numerical simulation models.

**Figure 6:**
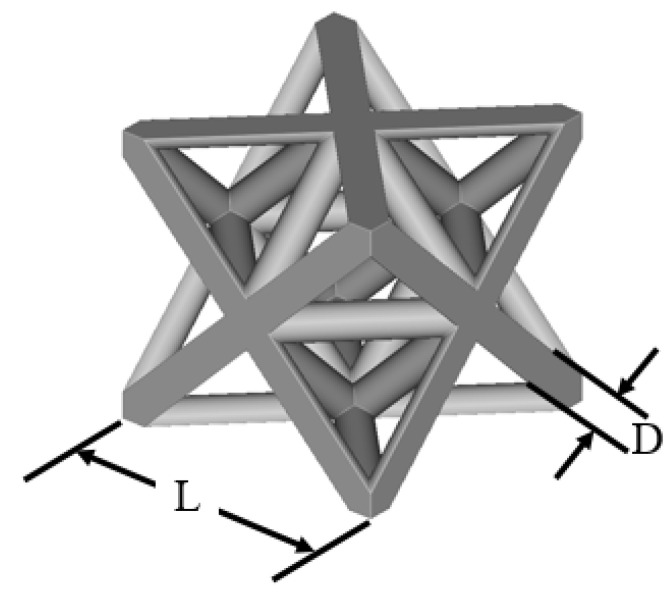
Octet truss unit cell with length (L) and strut diameter (D) lattice dimensions.

**Figure 7:**
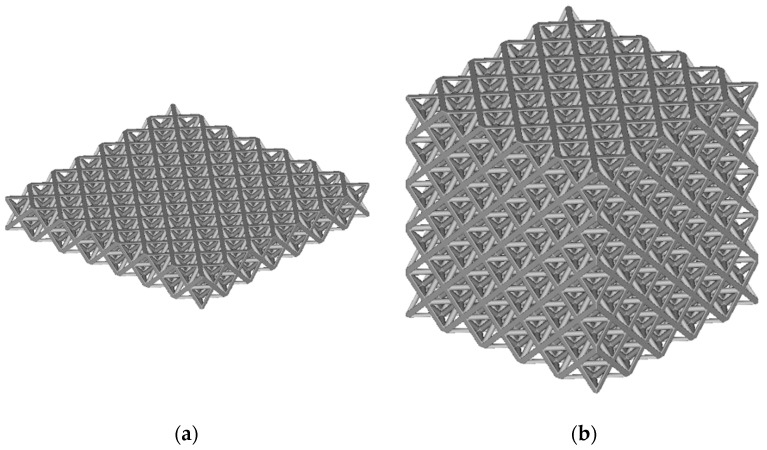
(**a**) 7 × 7 array of octet truss unit cells; (**b**) 7 × 7 × 7 array of octet truss unit cells.

The octet truss unit cell shown in Figure 6 is used as the basic building block in this study. All lattice design work done in this study used nTop Platform software from nTopology. The parameter *L* in Figure 6 refers to the unit cell’s length. For this study, *L* = 12 mm. The bounding box length, depth, and height are equal. The parameter *D* refers to the diameter of the cylindrical beams that make up the unit cell. Figure 7a shows a 2D array of octet truss unit cells with 7 unit cells in each of the width and depth directions (i.e., a 7 × 7 array). Figure 7b then shows a 3D 7 × 7 × 7 unit cell array. The 2D array in Figure 7a represents one layer of the 3D lattice block in Figure 7b. Given that *L* = 12 mm, the overall dimensions of the 7 × 7 × 7 unit cell array shown in Figure 7b is 84 mm × 84 mm × 84 mm.

### 2.1. Numerical Simulation

**Figure 8:**
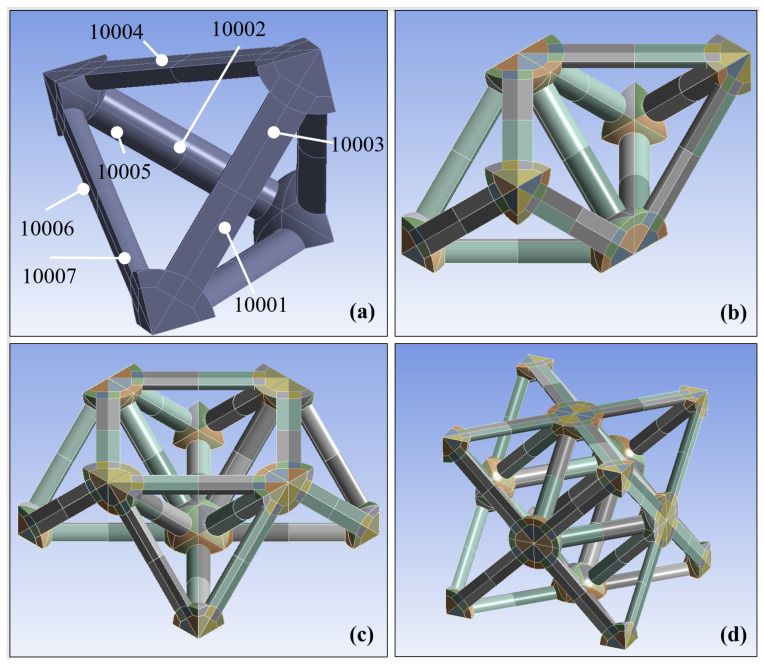
CAD Modeling an octet truss unit cell for numerical simulation; (**a**) six trusses that form a tetrahedron; (**b**) tetrahedron mirrored about the y-axis; (**c**) double tetrahedron from (**b**) mirrored about the x-axis; (**d**) half-call from (**c**) mirrored about the z-axis to form the octet truss unit cell.

**Figure 9:**
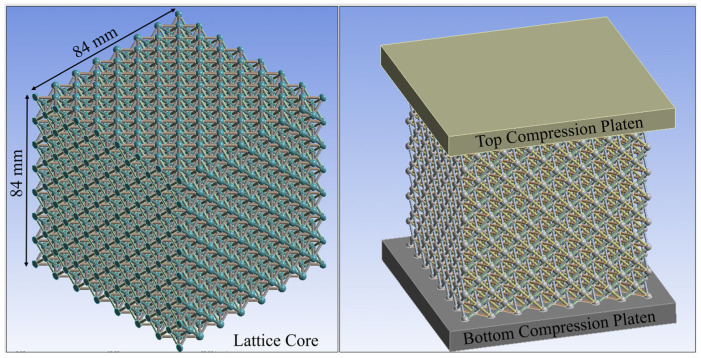
Digital twin of a 7 × 7 × 7 octet truss compressive test setup (**Left**) without compression testing plates, and (**Right**) with compression loading plates.

3D models of the octet truss lattice structure studied in this research were created using Ansys Mechanical and SpaceClaim version 2021.1.0.11221 software (Canonsburg, PA USA). Each truss was modeled as an independent body with its own orientation in 3D space. Six of these trusses were joined at their nodes to form a tetrahedron as seen in Figure 8a. This tetrahedron was then mirrored three times about the X, Y, and Z axes as shown in Figure 8b–d to form one octet truss unit cell. As mentioned previously, the unit cell bounding box dimensions were *L* = 12 mm per side. This unit cell was then patterned into a 7 × 7 × 7 3D array to form the lattice block shown in Figure 9. Seven unit cells are chosen in each direction to limit edge effects of unbounded cells on the outer faces of the array.

**Figure 10:**
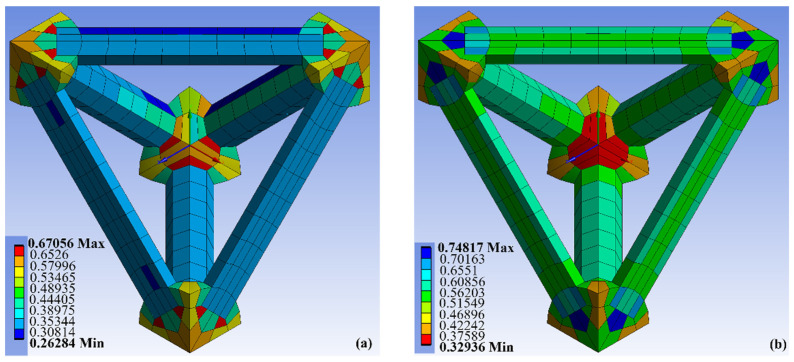
(**a**) Mesh element quality, where values closer to 1 are desired; (**b**) Mesh skewness, where values closer to 0 are desired.

Ansys Mechanical software was used to mesh the unit cell model. The total number of nodes in the basic block (i.e., points where mesh edges touch) was 1006, and the number of mesh elements per unit cell was 600. The tetrahedron was meshed using hexagonally shaped elements, as they generally provide more accurate results during large deformations. Mesh element quality (i.e., how close each mesh element is to a tetrahedron) and skewness (i.e., how close the edge lengths in a mesh element are to being equal) analysis results are shown in Figure 10. Higher values of mesh quality and lower values of skewness are desired. In this case, the mesh produces reasonably good results for the beams, with somewhat lower quality at the locations where the beams join at the nodes. The settings used for mesh generation are given in Table 1. Readers interested in knowing meanings of the mesh settings are referred to the Ansys online Training Center [38].

Material Type 122_3D was chosen in the Ansys LS-DYNA software module as it combines orthotropic elastic behavior with Hill’s 1948 anisotropic failure theory [39]. Table 2 shows the material parameter values that LS DYNA requires the user to supply when using Material Type 122_3D. Readers interested in learning more about the meanings of each parameter are referred to the Ansys online Training Center [38]. Yield strength and Young’s modulus in the axial direction were obtained directly from the datasheet provided by the supplier. For the other parameters, literature on orthotropic properties of carbon fiber composites was consulted. According to the literature, Young’s modulus decreases by a factor of ≈4 in the transverse direction for aligned carbon fiber composites [40]. The shear modulus was determined from literature that closely matches the Young’s modulus of the material used in this study. Shear modulus in the transverse direction was assumed to be twice of that in the axial direction, based on data presented by Duan et al. [41].

**Figure 11:**
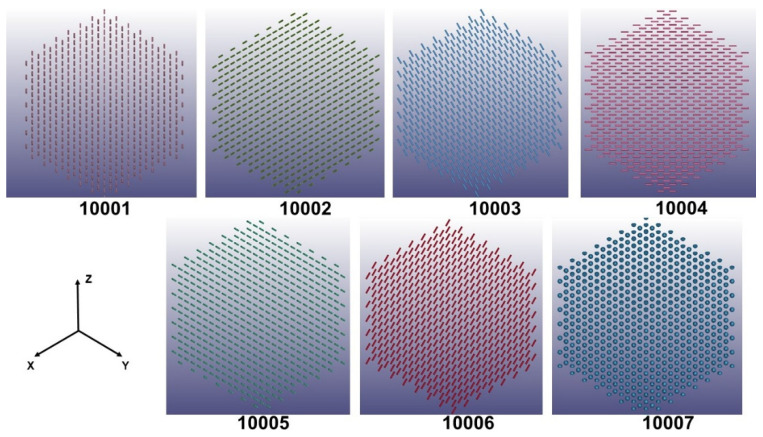
Local coordinate systems for bodies in the digital twin CAD model based on their orientation angle, and numbered from 10001 to 10006. All nodes were assigned the 10007 orientation.

In the octet truss unit cell, cylindrical beams exists in six unique orientations. A local coordinate system was therefore established for each of these orientations as shown in Figure 11 to capture the orthotropic properties of the composite lattice structure in the numerical simulation. The truss groups were assigned an orientation ID from 10001 to 10006, and the nodes were assigned 10007. Each of the orientations (10001 through 10006) refer to a specific beam orientation. Therefore, there are six principal orientation groups for the trusses, with all the nodes making up a seventh orientation group. Using a PC with 12 cores and 128 GB of memory, the Ansys simulation took 84 h.

### 2.2. Component Fabrication

**Figure 12:**
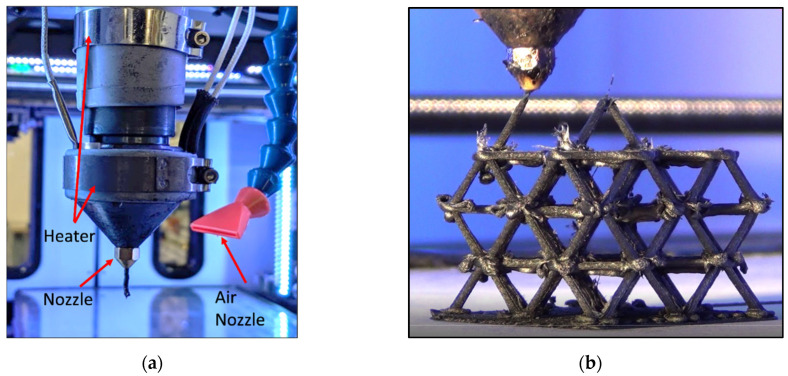
(**a**) Pellet extrusion system with air cooling used for non-planar ALE; (**b**) ALE octet truss structure fabrication.

All ALE fabrication was performed using a Titan Robotics Cronus FFF machine. This machine is equipped with a high temperature extrusion head which accepts pellet-based feedstock material. ABS-based chopped carbon fiber reinforced pellets with 20% carbon fiber loading from Sabic (LNP THERMOCOMP AC004) were used as the feedstock material. The system allows gas cooling of the extrudate material through a directed air nozzle seen in Figure 12a using the M46 (on) and M47 (off) g-code commands. A 1.2 mm diameter extrusion nozzle was used, and die swell of the extruded material resulted in struts having diameters of approximately 1.5 mm to match the 1.5 mm diameter struts analyzed in the FEA model. The extruder head travel speed during printing of each strut was 300 mm/min. An extrusion nozzle temperature of 280 °C was used for the carbon-filled ABS.

**Figure 13:**
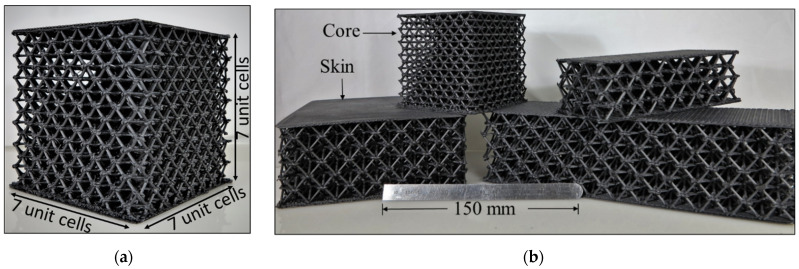
(**a**) Lattice block used for compression testing with 7 unit cells repeated in the X, Y, Z directions. (**b**) The ALE process used to fabricate lattice block in different unit cell configurations and shapes.

Figure 13a shows a printed 7 × 7 × 7 unit cell used for compression testing. This printed structure weighs 571 g and has a relative density (ratio of solid material volume to bounding box volume) of 17%. Octet truss structures having various unit cell sizes and replications are shown in Figure 13b.

### 2.3. Mechanical Testing

Three octet truss specimens were compression tested using an MTS 45-G universal testing machine. The quasi-static compression crosshead travel speed was 0.838 mm/s. Each specimen was compressed to 50% of its original specimen height.

## 3. Results

### 3.1. Mechanical Test Results

**Figure 14:**
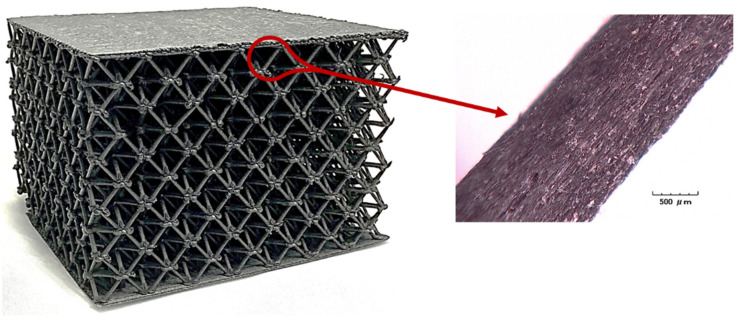
Close-up view of smooth continuous diagonal strut with axially aligned carbon fibers.

Figure 14 shows a lattice cube printed via the ALE approach as previously described. The inset photo shows a diagonal beam that was fabricated via the ALE approach. Rather than being a lamination of dozens of layers of material, the beam is one single smooth and continuous extrusion that eliminates all polymer layer interfaces within the beam. The image further shows the high degree of fiber alignment with the longitudinal axis of the cylindrical beam. This fiber alignment is conducive to high bending stiffness and tensile strength.

**Figure 15:**
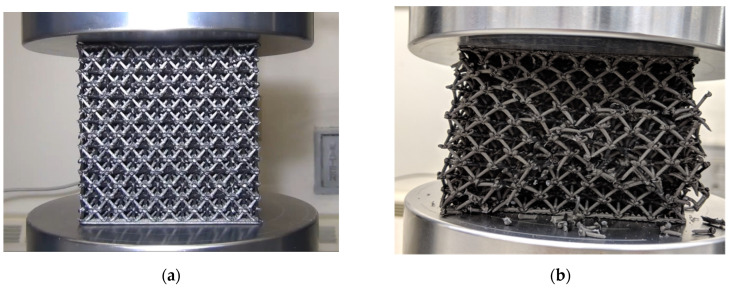
Compression testing of Octet Truss specimens on an MTS universal testing machine (**a**) before compression and (**b**) after compression.

Figure 15 shows a lattice structure at the onset of compression testing (Figure 15a) and then during compression testing after initial failure (Figure 15b). In all cases, the lattice structures failed at the nodes during compression testing. Trusses separated from other trusses in the network, starting approximately half way up the vertical height of the structure. From there, the failure started propagating to other layers. The trusses themselves did not fail by buckling or other failure mechanisms.

**Figure 16:**
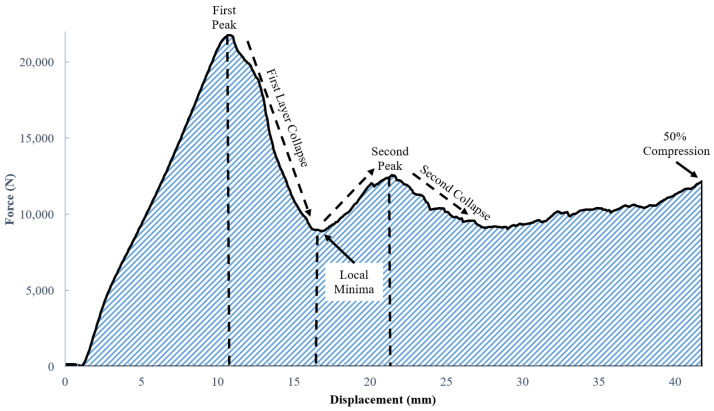
Results of quasi-static compression testing on a 7 × 7 × 7 unit cell octet truss specimen.

Figure 16 presents the force-displacement curves obtained from quasi-static compression of a representative specimen. The average peak load across all tested samples was 20.94 +/− 0.74 kN, and the average displacement at peak load was 10.01 +/− 1.05 mm. The first peak load in the Force-Displacement curve in Figure 16 occurs at 21,773 N with a displacement of 10.85 mm. The height of each of the 7 individual layers in the lattice structure is 12 mm, hence the 10.85 mm compression distance at peak loading occurs very close to the height of one layer of octet truss cells. As compression continues beyond 10.85 mm, the recorded load drops to a local minima value of 8894 N at a displacement of 16.97 mm. This drop in measured load occurs as individual struts within a layer begin to fracture at the nodes where they meet, thus greatly weakening that layer until the entire layer collapses. The rise in load from the 8894 N local minima up to the second peak represents the range where the compression platen has moved far enough down that the remaining layers then begin to carry load. The measured force then rises a second time up to the second peak in Figure 16. Beyond the second peak, the structure again experiences failure at the nodes leading to another collapse.

The hatched area under the Force versus Displacement curve in Figure 16 represents energy absorbed by the structure during quasi-static compression. This area was measured to be 463.2 N mm (= 0.4632 J) using a piecewise integration of the raw Force versus Displacement data in Excel up to 50% compression. Specific energy absorption (SEA) is equal to energy absorbed divided by the mass of the lattice structure. The sample used in Figure 16 had a mass of 571 g, giving an SEA value of 0.8111 J/g. Specific energy absorption is one of several useful metrics that can be used to estimate energy absorption potential as the size of the structure increases or decreases.

Force-Displacement plots may be converted to Stress–Strain plots, where the stress values are equal to the force divided by cross-sectional area of material that the load is applied to. The lattice block used in this study had nominal width and depth dimensions of 84 mm × 84 mm, giving an overall cross-sectional area of 7056 mm^2^. Lattice blocks contain a large percentage of void space though, and it is therefore not appropriate to use 7056 mm^2^ as the cross-sectional area for purposes of computing stress. Instead, the average cross-sectional area of the lattice structure (*A_XC_*) is computed as the volume of solid material in the lattice structure array (*V_LS_*) divided by the height of the lattice structure array (*L_LS_*):

(2) $$A_{XC}=\frac{V_{LS}}{L_{LS}}$$

For this example, *V_LS_* is measured to be 101,051 mm^3^ using nTopology, and *L_LS_* is 84 mm. Using Equation (2), *A_XC_* = 1203 mm^2^. The relative stress ($\sigma_{r}$) is then computed by dividing the measured force (*F*) values by the lattice structure’s average cross-sectional area:

(3) $$\sigma_{r}=\frac{F}{A_{XC}}$$

Strain is computed the conventional way by dividing the change in length at any given instant in time (ΔL) by the original length ($L_{0}$):

(4) $$\varepsilon =\frac{\Delta L}{L_{0}}$$

**Figure 17:**
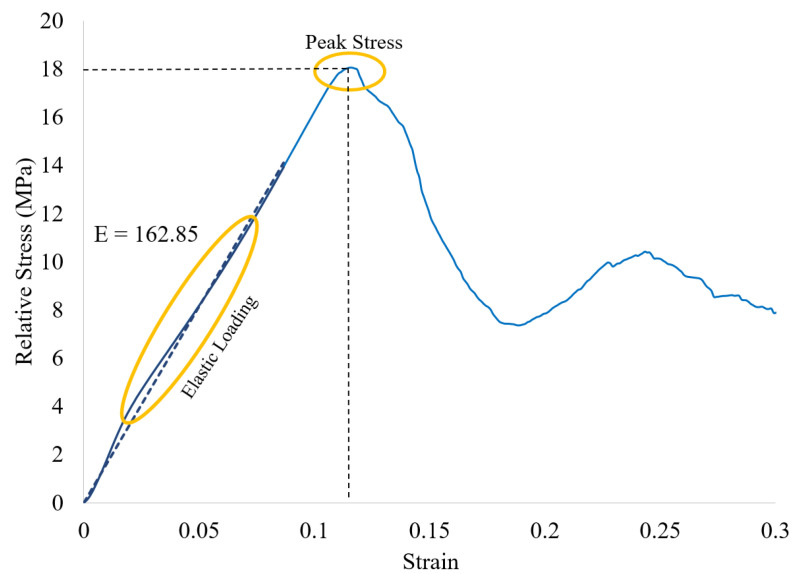
Relative stress–strain curve obtained for the octet truss lattice from Figure 17.

Using Equations (2)–(4), relative stress–strain curves may be plotted. Figure 17 shows the relative stress–strain curve that is derived from the force-displacement curve in Figure 16. The compressive strength (peak stress) value is 18.1 MPa for this sample. The average compressive stress across all samples was 17.4 +/− 0.6 MPa. A linear trend line for the elastic loading region in Figure 17 is plotted as a blue dashed line. The slope of this of this line (*E* = elastic modulus) is computed to be 162.85 MPa. The average modulus across all samples was 163.6 +/− 15.1 MPa. The significance of these properties in comparison with other studies in the literature is discussed in Section 4.

### 3.2. FEA Simulation Results

**Figure 18:**
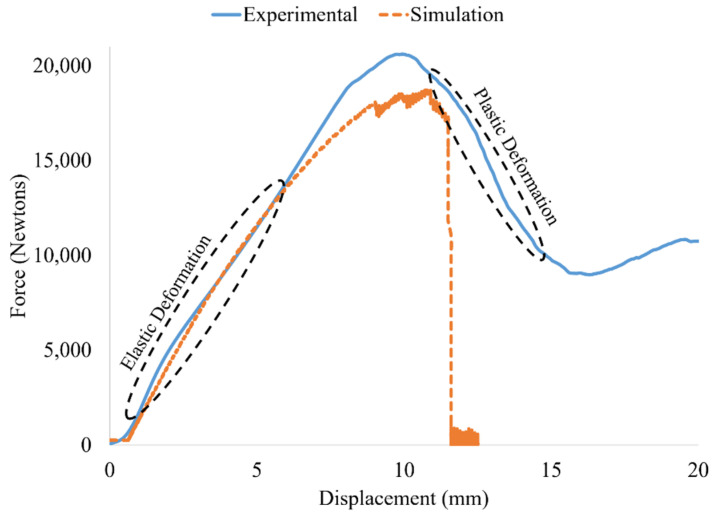
Comparison of Force vs. Displacement curve for average experimental data of three specimens and numerical simulation.

Figure 18 compares the load–displacement curve for the Ansys simulation predictions with the average experimental results across all samples (blue curve). The elastic (linear) deformation region of the Ansys curve aligns very well with the experimental data. As the two curves diverge from linearity into the plastic deformation region, numerical simulation is seen to have slightly under-predicted the peak load for the printed octet truss structures by ≈10%. The experimental data shows behavior more representative of a ductile material, which is typical of engineered lattice structures in which one layer of unit cells begins to fail.

The Ansys simulation results plotted in Figure 18 exhibit characteristics of a more brittle material with sudden catastrophic failure. This is attributed to the fact that in the simulation model, only the top and bottom layers of octet unit cells had contact regions defined with the compression platens. Once the top layer of unit cells failed, the next layer of unit cells did not have contact regions defined with the platen. The simulated loads were therefore not transmitted through lower layers once the top layer had completely failed. Defining contact regions between the platen and each layer of unit cells is possible, however, it would be extremely computationally expensive. The strong agreement between simulated and actual behavior in the elastic deformation region is of considerable value for applications in which the structure is designed to avoid failure. For high strain rate (impact) energy absorption applications where it is known that the structure will fail, full contact region definitions would be required.

Another challenge in improving on the 10% difference between simulated and physical test results for peak loading has to do with obtaining accurate bond strengths in the 3D printed nodes. All failures in physical testing occurred at the nodes. No instances of fracture within the smooth continuous extruded beams with aligned carbon fibers were observed. The nodes where beams come together are clearly the weak link within the ALE lattice structures. Obtaining more accurate node bond strengths is therefore critical to further improving simulation results. It must be emphasized, however, that a 10% deviation between predicted and actual peak loads for additively manufactured lattice structures is generally quite good.

**Figure 19:**
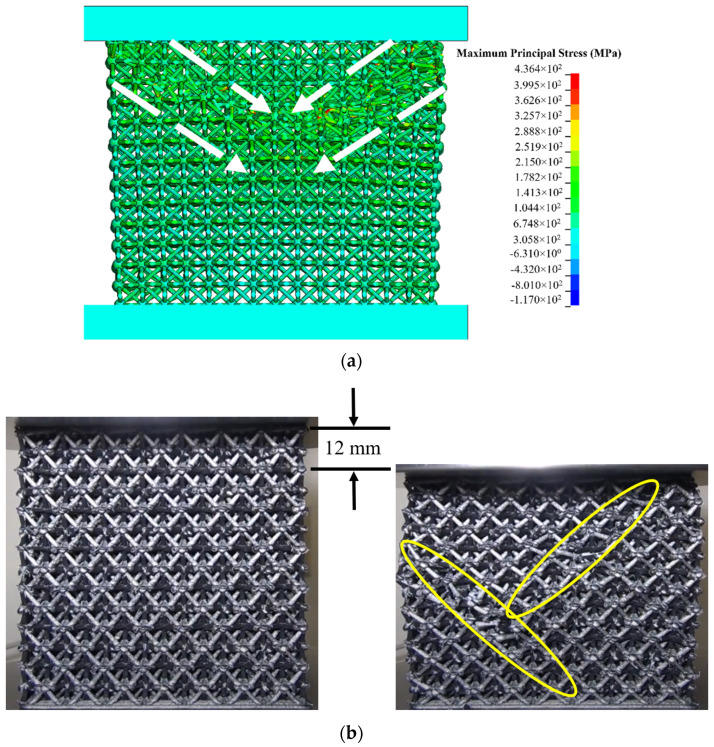
(**a**) First Principal Stress plot in the octet truss at the onset of failure; (**b**) Lattice structure prior to compressive load (left) and lattice structure after 12 mm of compression.

Figure 19a shows a 1st Principal Stress plot of the lattice block one time step in the simulation before failure was observed. White arrows have been added to the figure to show considerable deformation starting from the upper left and right corners beneath the upper compression platen. The deformation pattern has a pronounced 45° slanted orientation from the two upper corners towards the middle of the lattice block. For comparison with Ansys results, the left portion of Figure 19b shows a photograph of a lattice block taken before compressive loading. The right side of Figure 19b shows a photograph after 12 mm of compression. This closely maps to the first loading peak in Figure 16. The yellow ellipses in Figure 19b show the diagonals where struts have just started failing at the nodes. The diagonal failure line on the right half of the sample originate from the top right corner of the sample, whereas the diagonal failure line on the left half of the sample originates slightly below the upper left corner of the sample. The predicted failure mode from the Ansys model agrees reasonably well with experimental observations. It is noted that the failure mode for all tested samples was consistent with that seen in Figure 19b.

**Figure 20:**
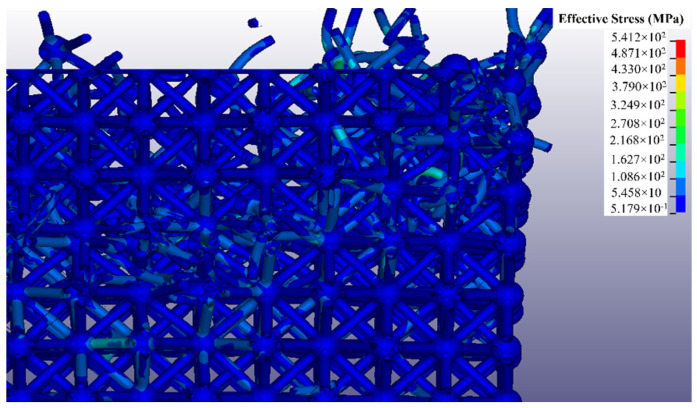
Cross section of the octet truss block sectioned from the center.

Figure 20 shows a zoomed in cross-sectional view of the top right corner of the Ansys model well after structural collapse has begun. The top compression platen is hidden from view in this figure in order to allow the broken struts to be seen. Broken nodes and struts are seen flying away from the structure primarily along the diagonal slant from the upper right corner. Again-this behavior is consistent with experimental results. A key observation from both physical experimentation and Ansys simulation results was that failure always occurred at the nodes. Beams did not fracture from excessive buckling.

## 4. Discussion

**Figure 21:**
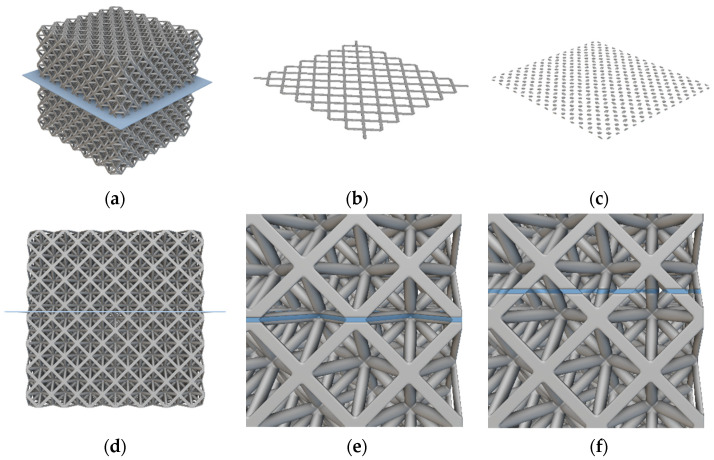
Cross-sectional slice images; (**a**) lattice block with slice plane, (**b**) cross-sectional slice at a height of z = 42 mm, (**c**) cross-sectional slice at a height of z = 42.3 mm, (**d**) front view of lattice block slice plane, (**e**) close-up view of slice volume (blue region) at z = 42 mm, (**f**) close-up view of slice volume (blue region) at z = 42.3 mm.

While the average cross-sectional area was used to compute relative stress, the actual cross-sectional area varies depending upon the vertical location in the sample where a cross-sectional slice is taken. Figure 21 very clearly illustrates this phenomena using the 84 mm × 84 mm × 84 mm octet truss lattice block studied in this work. The cross-sectional slice shown in Figure 21b was taken at a height of 42 mm up from the bottom of the lattice sample. The cross-sectional slice shown in Figure 21c was taken at a height of 42.3 mm up from the bottom of the lattice sample. In Section 3, the average cross-sectional area used to determine relative compressive stress was computed to be 1203 mm^2^, however, the cross-sectional area of the 2D lattice slice shown in Figure 21b is 2275 mm^2^, and the cross-sectional area of the slice in Figure 21c is 981 mm^2^. Instead of using average cross-sectional area to compute relative compressive strength, if the peak measured load of 21,773 N from Figure 16 were instead divided by the 981 mm^2^ cross-sectional area from the slice in Figure 21c, then the relative compressive strength of the sample at that height would be 22.2 MPa. That is well above the average compressive stress.

This analysis highlights the fact that actual compressive stress in a lattice structure varies depending upon vertical location within the sample. Figure 21f shows that the vertical planes where cross-sectional areas are lowest, and hence areas where compressive stress is the highest, occur in the middles of the beams between where they converge at the nodes. Conversely, the lowest compressive stress occurs in the layers where beams converge at the nodes (i.e., Figure 21b,e). Despite the highest compressive stress taking place in the middles of beams and away from nodes, no evidence of beam fracture/failure was observed in testing. This further underscores two things. First, the high beam stiffness resulting from alignment of carbon fibers within the beams was extremely effective at preventing failure due to buckling of diagonal struts or tensile fraction of horizontal struts. Second, the fact that the lattice structures failed at the nodes where relative compressive stress was lowest suggests a strong need for future research on increasing node strength with the ALE process. This is discussed in Section 5.

Since there is very little published research on mechanical properties of lattice structures fabricated with the ALE process, Table 3 presents the relative compressive strength and Young’s modulus of truss lattice structures fabricated in other related research efforts. These lattice structures have been made with different manufacturing methods, however, each one uses a polymer matrix composite material. Liu et al. [37] is the one entry that used volumetric ALE to fabricate lattice structures. However, Liu’s mechanical properties were based on a BCC lattice geometry rather than the octet truss studied in this work. The relative compressive strength of 17.4 MPa achieved in this work is the highest value among the composite truss lattice structure comparisons. Of particular note is the 162.8 modulus value in the present work. This strongly confirms the original hypothesis that alignment of carbon fibers with the longitudinal axis of each beam would results in very high stiffness of the structures. The ALE approach to construction of truss lattice structures with aligned carbon fibers therefore offers opportunities for putting lattice structures in end-use parts.

## 5. Conclusions and Future Recommendations

This paper has presented an Axial Lattice Extrusion (ALE) process for fabrication of high strength lattice structures using carbon fiber polymer matrix composite materials. With traditional FFF in which printing is done one slice layer on top of another, each vertical and diagonal beam that makes up a lattice structure has dozens of weak interfaces between layers of small round or elliptical disks as illustrated in Figure 3. A major advantage of this approach is that the beams are instead produced in one single extrusion motion. ALE completely eliminates the weak interfaces between printed layers in the beams that are seen in previously published research that uses layerwise FFF to product lattice structures. As mentioned in Section 1, large scale parts used in transportation applications will have millions of these interfaces if traditional layerwise FFF printing is used.

By using chopper fiber reinforced feedstock material with the ALE approach, a second major advantage is realized. As seen in Figure 14, a very high degree of fiber alignment is seen in the ALE struts. This contributes to the strength and stiffness of the ALE structure. When compared with other published work on carbon fiber polymer lattice structures, the present work shows excellent relative compressive strength (17.4 MPa) and modulus (162.8 MPa) values.

One disadvantage with ALE identified in this work is the fact that the extremely strong and stiff beams that make up the lattice structure must still be joined together where they meet at the nodes. The nodes are the weakest links in the resulting structures. Note that this holds true whether the structure is fabricated via ALE or layerwise FFF. The primary focus for future research is therefore as follows:

- Modifications to the ALE print strategy will be studied to increase node bond strength via printing of additional material and/or modifications to nozzle temperature and speed at the nodes to enhance polymer entanglement.

An Ansys finite element modeling approach has also been presented that captures the orthotropic properties of the carbon fiber octet truss lattice structure. The Ansys model predicts failure of the structures via diagonal shear starting from the upper left and right corners. The experimentally observed failure closely mimicked the predicted shear failure patterns from the Ansys model. The Ansys modeling approach is therefore a useful starting point for analyzing more complex designs. One area where the Ansys model needs future work is in predicting properties beyond the yield stress of the material. Results indicated good agreement between experimental and simulated Force-Displacement behavior in the linear elastic region. The second recommendation for future research is as follows:

- Contact interfaces between the platens and all beams will need to be defined in order to improve accuracy beyond the yield stress point.

Aside from improving node bond strength and enhancing the Ansys model, several other future research efforts are recommended. The current work was focused on demonstrating the ability of ALE to (1) eliminate interfaces within beams and (2) align fibers within the beams.

- Future studies should focus on studying behavior of lattices at different relative density values by increasing or decreasing the length parameter (*L*) value of the the lattice unit cell.

Likewise, the present study focused on compression testing. Real-world applications often involve multiple loading conditions in which material may be subject to compressive, tensile, flexural, and/or shear forces.

- Future tests should assess properties in those different loading conditions.

Lastly, Figure 1 shows many different types of lattice unit cell geometries. The ALE process is well suited for fabrication of any of the open cell lattice types. Another future research topic is therefore as follows:

- Future research should explore properties of each unit cell type in order to gain insight into the loading conditions that each unit cell type are best suited for. In applications involving multiple loading conditions, blends of different lattice unit cell types within a single structure may ultimately be the ideal approach.

## Figures and Tables

**Table 1:** Basic mesh settings used in Ansys Mechanical.

Parameter	Units and Value
Element Order	Linear
Element Size	0.75 mm
Transition	Fast
Span Angle Center	Coarse
Initial Seed Size	Assembly
Bounding Box Diagonal	10.825 mm
Average Surface Area	0.59879 mm^2^
Minimum Edge Length	0.1829 mm
Error Limits	Aggressive Mechanical
Target Quality	Default (0.05)
Smoothing	Medium
Rigid Body Behavior	Dimensionally Reduced

**Table 2:** Material properties used in LS-Dyna for numerical simulation using material 122_3D.

Parameter and LS-Dyna Keyword Code	Value and Units
Yield strength in axial direction of truss (mat_sigx)	89 MPa
R mat_P1 (k_truss_)	3543
R mat_P2 (n_truss_)	0.8
R mat_P1_2 (k_node_)	716
R mat_P2_2 (n_node_)	0.8
Young’s modulus in axial direction (R mat_E_a_)	11.8 GPa
Young’s modulus in transverse direction (R mat_E_b_ and E_c_)	6 GPa
Shear modulus in axial direction (R mat_R_xy_)	3 GPa
Shear modulus in transverse direction (R mat_S_zx_ and T_xy_)	6 GPa
Material density	1.8 × 10^−9^ Mg/m^3^
Poisson’s ratio axial direction	0.4
Poisson’s ratio transverse direction	0.015

**Table 3:** Comparison of the present work with other existing truss lattice structures.

Reference Work	Matrix Material	Manufacturing Method	*σ_R_* (MPa)	E_r_ (MPa)
Xu et al. [42]	Carbon-PP	Reversible assembly	0.12	3.7
Schneider et al. [43]	Carbon-PET	Fold-cut	1.90	80.0
Liu et al. [37]	CFRP-PLA	ALE	1.24	27.7
Present Work	CFRP-ABS	ALE	17.40	162.8

## Data Availability

Lattice models and raw compression testing data is available upon request from the corresponding author.
